# Blue TiO_2_ nanotube arrays as semimetallic materials with enhanced photoelectrochemical activity towards water splitting

**DOI:** 10.3906/kim-2004-85

**Published:** 2020-12-16

**Authors:** Naeimeh Sadat PEIGHAMBARDOUST, Umut AYDEMIR

**Affiliations:** 1 Boron and Advanced Materials Application and Research Center, Koç University, İstanbul Turkey; 2 Department of Chemistry, College of Sciences, Koç University, İstanbul Turkey

**Keywords:** Blue TiO_2_nanotubes, cathodic polarization, self-doping, photoelectrochemical properties, water splitting

## Abstract

In the past years there has been a great interest in self-doped TiO_2_ nanotubes (blue TiO_2_ nanotubes) compared to undoped ones owing to their high carrier density and conductivity. In this study, blue TiO_2_ nanotubes are investigated as photoanode materials for photoelectrochemical water splitting. Blue TiO_2_ nanotubes were fabricated with enhanced photoresponse behavior through electrochemical cathodic polarization on undoped and annealed TiO_2_ nanotubes. The annealing temperature of undoped TiO_2_ nanotubes was tuned before cathodic polarization, revealing that annealing at 500 °C improved the photoresponse of the nanotubes significantly. Further optimization of the blue TiO_2_ nanotubes was achieved by adjusting the cathodic polarization parameters. Blue TiO_2_ nanotubes obtained at the potential of –1.4 V (vs. SCE) with a duration of 10 min exhibited twice more photocurrent response (0.39 mA cm^-2^) compared to the undoped TiO_2_ nanotube arrays (0.19 mA cm^-2^). Oxygen vacancies formed through the cathodic polarization decreased charge recombination and enhanced charge transfer rate; therefore, a high photoelectrochemical activity under visible light irradiation could be achieved.

## 1. Introduction

Recent research activities on water splitting have focused on photocatalyst (PC) and photoelectrochemical (PEC) systems. Due to the fact that in PC systems the generated oxygen and hydrogen are not immediately separated, the PEC system has attracted significant interest. Illumination to a conductive electrode like TiO_2_ leads to the generation of electron and hole pairs [1,2].

Photoelectrochemical (PEC) water splitting for H_2_ generation has been used extensively to prevent environmental pollution [3–5]. PEC systems effectively separate the charge pairs into an anode and a cathode via absorbing light, and photochemical conversions can be achieved through the reduction of water to hydrogen [6,7]. TiO_2_ is one of the most attractive materials for storage and conversion of energy exemplified as a photoanode for PEC water splitting [8–10], CO2 reduction [11], and Li-ion battery [12] due to its nontoxicity, low cost, chemical stability, strong optical absorption, and photocorrosion resistance.

Among the different morphologies of TiO_2_ nanostructures, a lot of effort has been devoted to TiO_2_ nanotubes prepared by electrochemical anodization as efficient photoanodes for PEC cells due to simple fabrication, high surface area, one-dimensional nanostructure, and high orientation [13–15]. The well-ordered TiO_2_ nanotubes provide enhanced charge separation and effective charge carrier transportation by decreasing the distance of charge carrier to electrolyte and increasing the electrode-electrolyte interfaces [16].

However, TiO_2_ has been undesirable due to its weak electroconductivity and high levels of trap states [17,18]. The photocatalytic efficiency of TiO_2_ is substantially restricted by its large bandgap energy and fast electron-hole recombination. The large bandgap of TiO_2_ leads to the utilization of only the UV portion of light (only 4% of light). Therefore, bare TiO_2_ suffers from weak light-harvesting and strong surface reflection [19–21].

To enhance light absorption, a lot of research activities have been focused on, for example, compositing via semiconductors with a small bandgap [22], surface plasmon resonance of noble metal [23,24], dopant-free bandgap narrowing by improving TiO_2_ morphology and electronic structure [25,26], and intercalation of doping elements [27–30]. There are many efforts on modifying the valance band of TiO_2_ by using nonmetals like N [31], S [32], and C [33]. The P states of these ions create impurity states above the valance band, therefore it shifts the valance band edge to higher levels and narrows the bandgap for harvesting the visible light [34]. On the other hand, in metal doping, cations fill the Ti sites and they introduce midgap electronic states inside the bandgap of TiO_2_ [35,36].

Although metallic and nonmetallic element doping creates donor or acceptor states in the bandgap, dopants act as carrier recombination centers and decrease the efficiency of PEC [37]. Since the dopants introduce impurity states inside the TiO_2_ bandgap, they lead to crystal instability and increase of charge carrier trapping [38]. To overcome these limitations, self-doping (reductive doping) has been introduced as a new strategy for increasing PEC efficiency. Several methods have been introduced to develop self-doped TiO_2_ such as hydrogenation [39] and chemical and electrochemical reduction of TiO_2_ [40]. As a result of reductive doping, the intrinsic defect concentrations are changed in the semiconductor. During the self-doping process, oxygen vacancies and Ti^3+^ are formed, which can strongly enhance the PEC properties [41].

Narrowing the bandgap by means of oxygen vacancy states leads to an enhancement of light absorption followed by a color change to blue [42,43]. TiO_2_ nanotubes exhibit an electrochromism process under a reduction environment and it could be turned to blue or black. The creation of Ti^3+^ centers is accompanied by proton intercalation in the nanotubes [44].

Oxygen vacancies, as shallow donors, play a significant role in TiO_2_ electronic properties [45]. Oxygen vacancies and Ti^3+^ improve the light absorption of TiO_2_ by separation of charge carriers. In fact, oxygen vacancies introduce impurity levels of 0.5–0.7 eV beneath the conduction band, therefore the electronic states could be generated inside the TiO_2_ band gap [41,46]. This results in enhanced charge transport behavior and light absorption. In fact, via self-doping, TiO_2_ semiconductor behavior of TiO_2_ could be turned into an almost semimetallic behavior [47].

It is worth noting that during the reduction, the voltage should be optimized to prevent the evolution of excessive hydrogen [48] in this system. After all, developing a strategy to synthesize TiO_2_ nanotubes with narrowing bandgap and high photoelectrochemical application is still a great challenge.

In this study, self-doped TiO_2_ nanotube arrays (NTAs) are fabricated by using a facile cathodic polarization method producing an effective photocurrent for water splitting applications.

## 2. Materials and methods

An electrochemical anodization process was used to produce TiO_2_ nanotube arrays. A commercial pure titanium sheet (grade 2, 0.6 mm) was prepared for this purpose as the anode material in the anodization process. Titanium samples were cut in dimensions of 1 × 2 cm^2^ and ground step by step up to 1200 grid. For more smoothing, mechanical polishing with Al2O3 slurry (0.05 µm powder) was applied. The samples were then cleaned in acetone and ethanol for 15 min in an ultrasonic bath. The electrochemical anodization was carried out in two steps. In both steps, anodization was performed at the voltage of 60 V (high power DC power supply KXN6010D) for 4 h at 30 °C and in ethylene glycol (EG), 0.15 M NH4F, and 3 vol.% DI water solution. Cylindrical stainless steel was used as the cathode material. During anodization, the electrolyte was agitated by using a magnetic stirrer.

The TiO_2_ nanotube layer formed at the first step was ultrasonically removed in distilled water to expose the fresh surface of Ti. Then, the second anodization step was performed on this fresh surface. As-anodized samples were annealed at three different temperatures of 480, 500, and 520 °C by heating at the rate of 1.5 °C/min for 4 h. Subsequently, they were left in the furnace to cool down.

Blue TiO_2_ nanotubes were produced by using cathodic electrochemical polarization in a three-electrode system with a workstation (AUTOLAB). The TiO_2_ nanotube samples annealed at 500 °C were placed as a working electrode in the supporting electrolyte of 0.5 M Na_2_SO_4_. A platinum sheet (1.5 × 3 cm^2^) and a saturated calomel electrode were used as the counter and reference electrodes, respectively. The cathodic polarization process was performed at –1.2, –1.4, and –1.6 V for durations of 5, 10, and 20 min. After cathodic polarization, the blue TiO_2_ nanotubes were dried in an oven at 80 °C for 30 min.

The surface morphology characterization of TiO_2_ NTAs was performed by field emission scanning electron microscopy (FE-SEM; MIRA3FEG-SEM, TESCAN USA, Inc.,Warrendale, PA, USA). An X-ray diffraction technique [X’Pert PRO MPD (Malvern Panalytical Inc., Westborough, MA, USA)] employing Cu Kα radiation was used to characterize the phases and their purities. The chemical composition of metal oxide samples was characterized by X-ray photoemission spectroscopy (XPS) (SPECS EA 300 equipped with Al monochromatic anode). To examine the electrochemical response of TiO_2_ NTAs before and after doping, cyclic voltammetry (CV) (AUTOLAB) was utilized in the dark and at a scan rate of 100 mV.s−1.

The PEC performances of the undoped and doped TiO_2_ NTAs were evaluated in a three-electrode configuration consisting of TiO_2_ NTAs as the working photoelectrode, Ag/AgCl as the reference, and Pt rod as the counter electrode. The measurements were conducted in the supporting electrolyte of 0.5 M Na_2_SO_4_ using potentiostat/galvanostat (AUTOLAB) under solar-like irradiation of Xe lamp at AM 1.5 (100 mW·cm−2). Linear sweep voltammetry (LSV) was performed at a scan rate of 100 mV.s−1. The chronoamperometric study was also carried out under chopped light irradiation (light on–off cycles: 20 s) at applied fixed bias of +0.5 V. Mott−Schottky measurements were performed at different applied DC potentials between −0.1 to 0.9 V and a frequency of 1000 Hz with a step potential of 0.05 V.

The electrochemical response of the pristine and blue TiO_2_ NTAs was investigated by electrochemical impedance spectroscopy (EIS) studies in a conventional three-electrode cell employing an electrochemical workstation (AUTOLAB) under a supporting medium of 0.5 M Na_2_SO_4_. All EIS tests were performed at an open-circuit voltage and the frequency window differed from 100 kHz to 0.01 Hz with a 10 mV rms sinusoidal modulation.

## 3. Results and discussion

### 3.1. Morphology characterization

**Figure 1:**
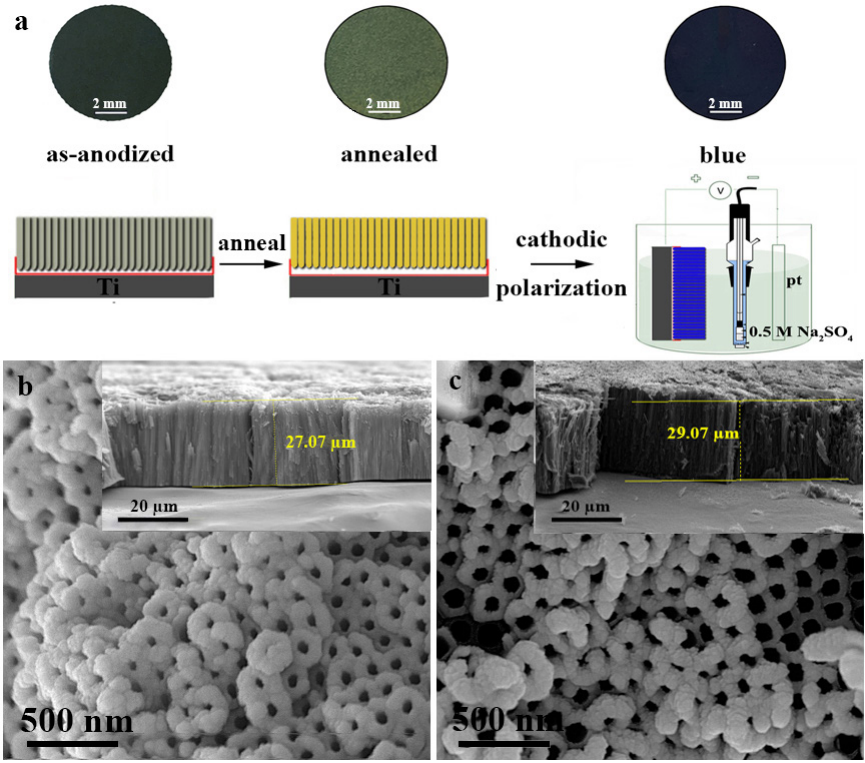
a) Macrophotograph of surface colors and process schematic of as-anodized, annealed (pristine) and blue TiO_2_NTAs and FESEM images of b) pristine and c) blue TiO_2_NTAs (the insets show the cross-section view of the nanotubes).

Figure 1 presents the process schematic, the macro photograph of surface colors, and FESEM images of the TiO_2_ NTAs. As shown in Figure 1a, the as-anodized TiO_2_ NTAs were grey and after annealing at 500 °C turned to yellowish-grey. As seen from the process schematic in Figure 1a, to produce blue TiO_2_ NTAs, the annealed TiO_2_ NTAs (as called pristine) were reduced under the cathodic polarization process. The reduction process has occurred during the cathodic polarization and blue coloration of the annealed TiO_2_ NTAs was observed due to electrochromism similarly found in transition metal oxides such as TiO_2_ [49]. In fact, the electrochromic effect has occurred owing to the reduction of Ti^4+^ to Ti^3+^ together with proton intercalation. During the cathodic polarization in Na_2_SO_4_ supporting electrolyte, Ti^4+^ is reduced to Ti^3+^ after applying a negative potential. The accumulation of electrons has occurred on the semiconductor film and charge compensation was achieved by a proton as follows [50]:

Ti^4+^+ + e^-^ + H^+^ = Ti^3+^ + H+

The Ti^3+^ defects (or oxygen vacancy states) are formed because of injected electrons below the conduction band. Introducing the oxygen vacancy states leads the semiconductor pristine TiO_2_ NTAs to transform into semimetallic blue TiO_2_ nanotubes [26]. Figures 1b and 1c show FESEM images of the surface and also cross-sections (the insets) of pristine and blue TiO_2_ NTAs, respectively. As shown in the figures, there is no difference between the surface morphologies of pristine and blue TiO_2_ NTAs. The reason is that both have the same anodizing time and annealing process. Furthermore, the electrochemical reductive process only changes electronic and defect levels of blue TiO_2_ NTAs, which has no obvious effect on the shape and length of the tubes.

**Figure 2:**
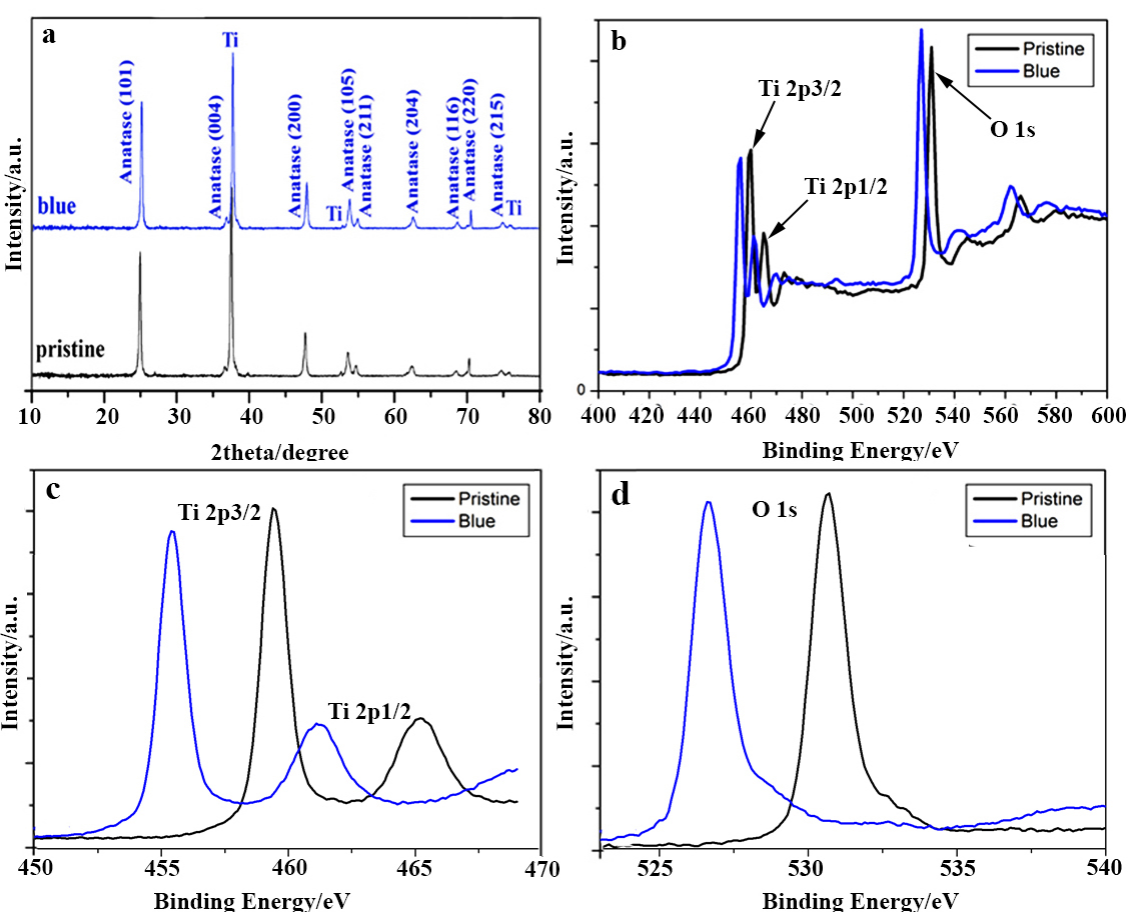
a) XRD patterns (anatase TiO2JCPDS card no. 21-1272) and b) XPS spectra, c and d) high-resolution XPS spectra of pristine and blue TiO_2_NTAs.

Figures 2a and 2b show XRD and XPS patterns of the pristine and blue TiO_2_ NTAs. Based on the XRD patterns (Figure 2a), the strong peak at 25.31 demonstrates that both the pristine and the blue TiO_2_ NTAs are completely crystalized and consist of mostly pure anatase phase. Furthermore, there are no clear differences between the XRD patterns of pristine and blue TiO_2_ NTAs, as their peaks positions are the same. Therefore, the phase structure is not affected by reductive doping and electrochromism. To investigate surface bonding, XPS spectrums of TiO_2_ NTAs are shown in Figures 2b–2d. From high-resolution XPS spectra for the Ti2p and O 1s regions shown in Figures 2c and 2d, it can be concluded that the binding energies of Ti2p and O 1s are shifted to lower energies. These negative shifts are due to the presence of Ti3p and oxygen vacancies compared to the pristine one. For pristine TiO_2_ NTAs, the binding energies of Ti2p3/2 and 2p1/2 XPS peaks are around 460 and 465 eV, respectively. These energies are typical for the Ti^4+^–O bonds of TiO_2_ [26,51]. On the other hand, from Figure 2c it can be clearly seen that in blue TiO_2_NTA, the Ti2p3/2 and 2p1/2 peak positions are shifted to lower energies. In blue TiO_2_ NTAs, the Ti2p3/2 and 2p1/2 peaks are centered at about 455 and 462 eV, respectively. This negative shift indicates the presence of oxygen vacancies and confirms the 2p3/2 and 2p1/2 peaks of Ti^3+^ [52]. The O 1s XPS spectra also display the same negative shift with a single peak at about 532 and 527 eV for pristine and blue TiO_2_ NTAs, respectively (Figure 2d). The XPS characterization findings offer oxygen vacancies that are intercalated into the TiO_2_ lattice during the cathodic polarization process.

To optimize the annealing temperature of pristine TiO_2_NTA, the annealing process was performed at three different temperatures of 480, 500, and 520 °C. Based on our previous work [53], the XRD patterns of the TiO_2_NTA annealed at 480, 500, and 520 °C showed only the anatase crystalline phase without detecting a rutile phase. Also, the XRD patterns revealed that pristine TiO_2_NTA annealed at 500 °C has a much stronger intensity ratio of the anatase (101) peak than those of pristine TiO_2_NTA annealed at 480 and 520 °C.

### 3.2. Photoelectrochemical measurements

#### 3.2.1. Annealing temperature

**Figure 3:**
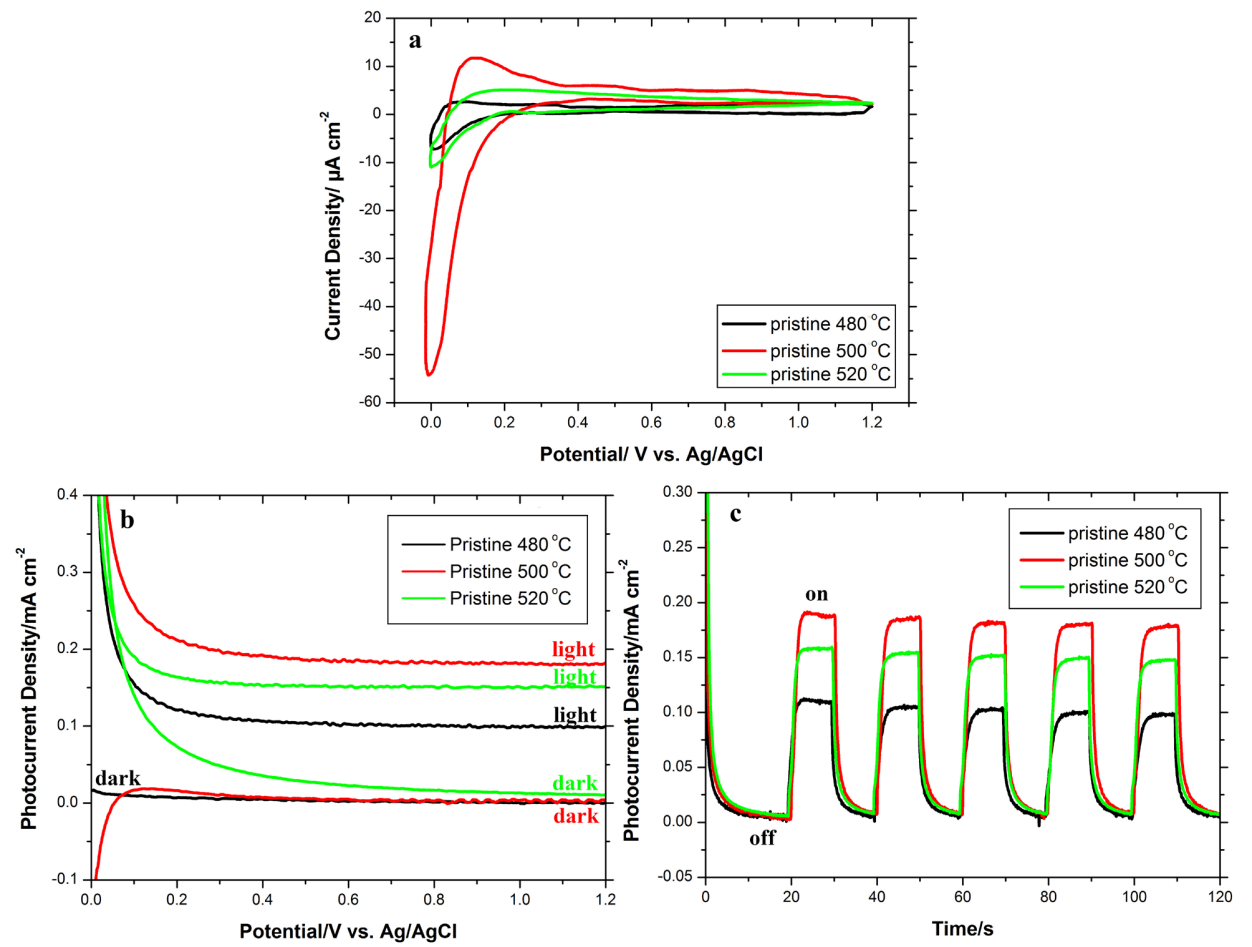
a) CV curves at a scan rate of 100 mV s^-1^ in the absence of light source, b) linear sweep voltammograms collected under 100 mW cm^–2^ illumination at a scan rate of 100 mV s^-1^, and c) photoresponse vs. time under chopped light irradiation at fixed +0.5 V applied bias of the pristine TiO_2_NTA annealed at different temperatures of 480, 500, and 520 °C.

The cyclic voltammetry (CV) test was performed in the dark on various annealed pristine TiO_2_ NTAsin order to scan all possible redox reactions on the surface and find the proper potential for the next photoelectrochemical investigations. Therefore, CV tests were performed in three electrode systems in 0.5 M Na_2_SO_4_ solution at a scan rate of 100 mV s–1. Figure 3a shows the related voltammograms. As seen from Figure 3a, with increasing the applied bias, the current density is enhanced because of water oxidation. In contrast, decreasing the applied bias reduces the current density owing to water reduction. The applied bias of chronoamperometry tests should be selected far enough from redox potentials. Figure 3a shows that +0.5 V could be the proper potential for photoelectrochemical investigations. It is worth noting that as shown in Figure 3a, the pristine TiO_2_NTA annealed at 500 °C possesses significantly higher current density due to anatase structure and generally anatase is much more conductive than amorphous TiO_2_.

To investigate the photoelectrochemical properties, the photocurrent densities of different pristine samples were measured by linear sweep voltammetry (LSV) in a three-electrode electrochemical system at a scan rate of 100 mV s–1 in the dark and under 100 mW cm^-2^ illumination. As shown in Figure 3b, all pristine samples revealed a very low dark current. No electrocatalytic water splitting occurs in the dark. Obviously, under illumination, the current density is enhanced and the pristine annealed at 500 °C shows higher photocurrent density indicating more effective charge separation. Figure 3c shows the chronoamperometry plots of pristine samples annealed at different temperatures under chopped irradiation and applied voltage of +0.5 V. A good photoresponse can be seen in chopped light cycles in all samples. In the dark mode, the current values are almost zero, whereas upon illumination, the photocurrent rapidly increases to a steady-state value and significant photoactivity can be observed. This manner is repeated for all on/off cycles. From Figure 3c, the steady-state photocurrent of the pristine annealed at 500 °C (0.19 mA cm^-2^) is nearly 2 times higher than of pristine annealed at 480 °C (0.11 mA cm^-2^) and also nearly 1.2 times higher than of pristine annealed at 520 °C (0.16 mA cm^-2^), indicating a higher photoactivity of the pristine annealed at 500 °C. Therefore, the annealing temperature of 500 °C was selected for further investigation of the blue TiO_2_ NTAs.

#### 3.2.2. Cathodic polarization voltage

**Figure 4:**
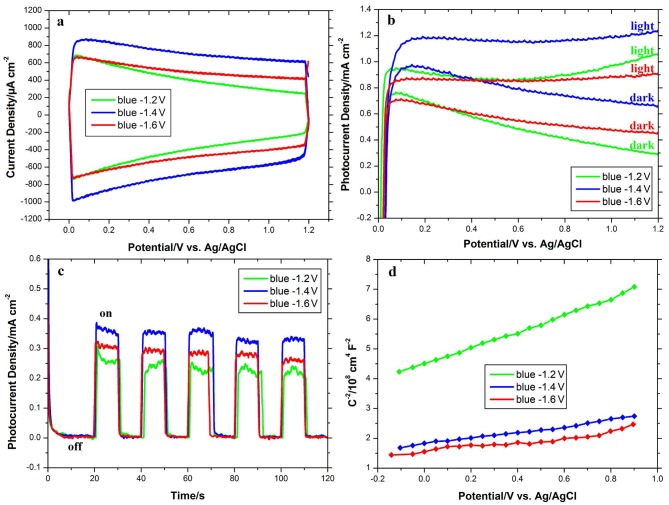
a) CV curves at a scan rate of 100 mV s^-1^ in the absence of light source, b) linear sweep voltammograms collected under 100 mW cm^–2^ illumination at a scan rate of 100 mV s^-1^, and c) photoresponse vs. time under chopped light irradiation at fixed +0.5 V applied bias of the blue TiO_2_NTAs synthesized at different negative cathodic polarization voltages of –1.2, –1.4, and –1.6 V.

The photocatalytic properties of blue TiO_2_ NTAs were evaluated by optimizing cathodic polarization parameters. For this purpose, cathodic polarization was performed at three different voltages of –1.2, –1.4, and –1.6 V to determine the proper synthesis voltage for photocatalytic application. Before recording the photoresponse of the samples, CV tests were performed in 0.5 M Na_2_SO_4_ solution at a scan rate of 100 mV s–1 in the dark to find a safe potential for the next photoelectrochemical investigations. Figure 4a shows the CV curves of blue TiO_2_ NTAs synthesized at different voltages. From Figure 4a, the CV curves of blue TiO_2_ NTAs have a rectangular shape characteristic, indicating a weak dependence of current density on potential because blue TiO_2_NTA reveals semimetallic behavior. In contrast, a strong dependence of current density on potential and consequently a triangular shape of the voltagram indicate semiconducting behavior (Figure 3a). As seen from Figure 4a, the blue TiO_2_ synthesized at –1.3 V reveals low current density and it still slightly retains the semiconducting feature due to the low density of Ti^3+^ states. Obviously, among the voltagrams in Figure 4a, the blue TiO_2_ at –1.4 V has higher current density owing to a higher level of Ti^3+^ states, indicating more efficient charge separation and transport in the sample with a high level of Ti^3+^ states. Also, increasing the negative voltage up to –1.6 V leads to more reduction and evolution of H2, therefore the current is declined [26]. Figure 4b shows LSV curves of the blue TiO_2_ NTAs synthesized at different voltages. From Figure 4b, it is obvious that unlike the pristine TiO_2_ NTAs, the blue ones have a remarkable current response in the dark. The blue TiO_2_ NTAs have greatly enhanced electrical conductivity because of the high content of Ti^3+^ states, therefore it reveals an increased dark current. Also, the blue TiO_2_ NTAs synthesized at –1.4 V has a high photocurrent compared to that of others due to its high content of Ti^3+^ states. According to the chronoamperometry plots of the blue TiO_2_ NTAs (Figure 4c) at an applied bias of +0.5 V and under chopped irradiation, it is clear that the steady-state photocurrent of blue TiO_2_NTA synthesized at –1.4 V (0.39 mA cm^-2^) is nearly 1.5 times higher than of the blue TiO_2_ NTAs at –1.2 V (0.25 mA cm^-2^) and nearly 1.2 times higher than of the blue TiO_2_ NTAs at –1.6 V (0.31 mA cm^-2^).The Mott–Schottky method was used to estimate the Ti^3+^ states densities. Figure 4d shows Mott–Schottky plots of the blue TiO_2_ NTAs synthesized at different voltages. By calculating the slope of the Mott–Schottky plot, the donor density of blue TiO_2_ NTAs could be obtained. The Mott–Schottky plot is based on the square of capacitance (1/C2) against applying potential. The space charge capacitance Csc of a semiconductor is expressed as follows:

1CSC2=2εε0eND(E-Efb-KBTe)

where N_D_ is the donor density, ε0 is the dielectric permittivity of the vacuum (8.854 × 10–14 F.cm–1), ε is the dielectric constant of the semiconductor (31 for TiO_2_ anatase), e is the elementary electric charge, and KB is the Boltzmann constant. Based on the Mott–Schottky equation and the slopes of the plots in Figure 4d, the donor densities of the blue TiO_2_NTA synthesized at –1.2, –1.4, and –1.6 V are 1.71 × 10^21^, 4.89 × 10^22^, and 4.19 × 10^22^ cm^-3^, respectively. This indicates that the blue TiO_2_ NTA at –1.2 V has a lower donor density than the blue samples at –1.4 and –1.6 V and there are slight differences between the donor densities of the blue TiO_2_ NTAs at –1.4 and –1.6 V.

#### 3.2.3. Cathodic polarization time

**Figure 5:**
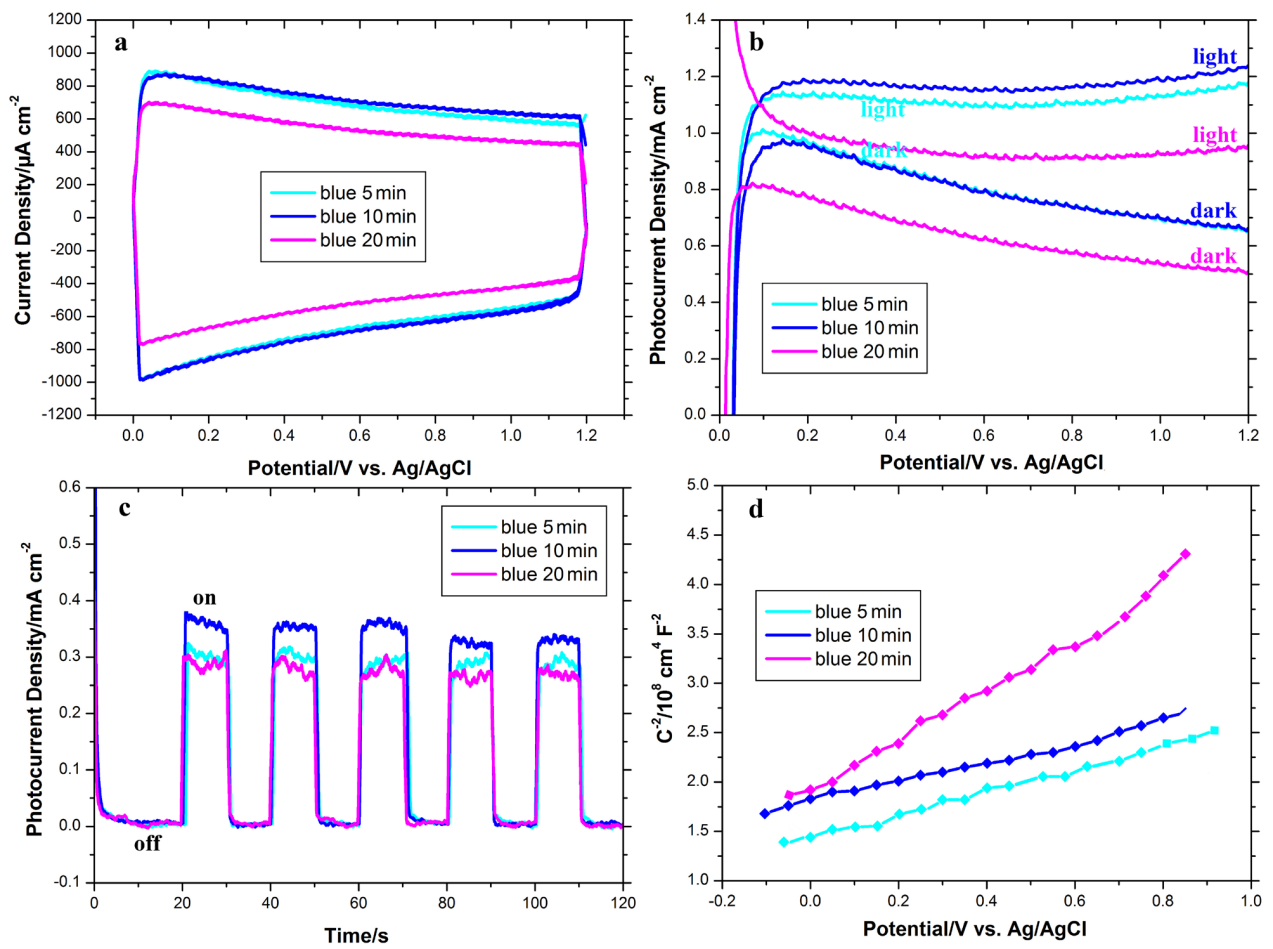
a) CV curves at a scan rate of 100 mV s^-1^ in the absence of a light source, b) linear sweep voltammograms collected under 100 mW cm^–2^ illumination at a scan rate of 100 mV s^-1^, and c) photoresponse vs. time under chopped light irradiation at fixed +0.5 V applied bias of the blue TiO_2_NTAs synthesized in different cathodic polarization times of 5, 10, and 20 min.

The duration of the cathodic polarization process for acquiring optimized blue TiO_2_ NTAswas also investigated. Figure 5a shows CV curves of blue TiO_2_ NTAs synthesized in 5, 10, and 20 min. As seen from the figure, the blue TiO_2_ NTAs synthesized in 5 and 10 min have the same current density, whereas the blue TiO_2_ NTAs synthesized in 20 min reveal lower current density. It is clear that increasing the reduction time of TiO_2_ NTAs up to 20 min enhances sample resistance and decreases conductivity due to the instability of oxygen vacancies and the destruction of TiO_2_ NTAs. To evaluate the photoelectrochemical properties, an LSV study was performed on blue TiO_2_ NTAs synthesized in different times of 5, 10, and 15 min; the results are shown in Figure 5b. As shown in Figure 5b, the blue TiO_2_NTA synthesized in 10 min has a higher photocurrent compared to that of NTA synthesized in 5 and 20 min, owing to its high content of Ti^3+^ states. In fact, more efficient charge separation and transport could have occurred in the high density of Ti^3+^ states. The chronoamperometry plots of the blue TiO_2_ NTAs synthesized at different times are also shown in Figure 5c. As seen from Figure 5c, the steady-state photocurrent of blue TiO_2_NTA synthesized in 10 min (0.39 mA cm^-2^) is nearly 1.3 times higher than of blue TiO_2_ NTAs synthesized in 20 min (0.3 mA cm^-2^) and nearly 1.2 times higher than of blue TiO_2_ NTAs synthesized in 5 min (0.34 mA cm^-2^). Obviously, the photoresponse of blue TiO_2_ NTAs was found to be weakened as the cathodic polarization time exceeds 10 min. The Mott–Schottky plots of blue TiO_2_ NTAs synthesized at different times are shown in Figure 5d. Based on the Mott–Schottky plots, the donor densities of the blue TiO_2_NTA synthesized in 5, 10, and 20 min are 3.35 × 1022, 4.59 × 1022, and 1.82 × 1021 cm^-3^, respectively. It can be clearly seen that when the cathodic polarization time extends to 20 min, the donor densities of the blue TiO_2_NTA decline.

#### 3.2.4. Optimized undoped and self-doped

**Figure 6:**
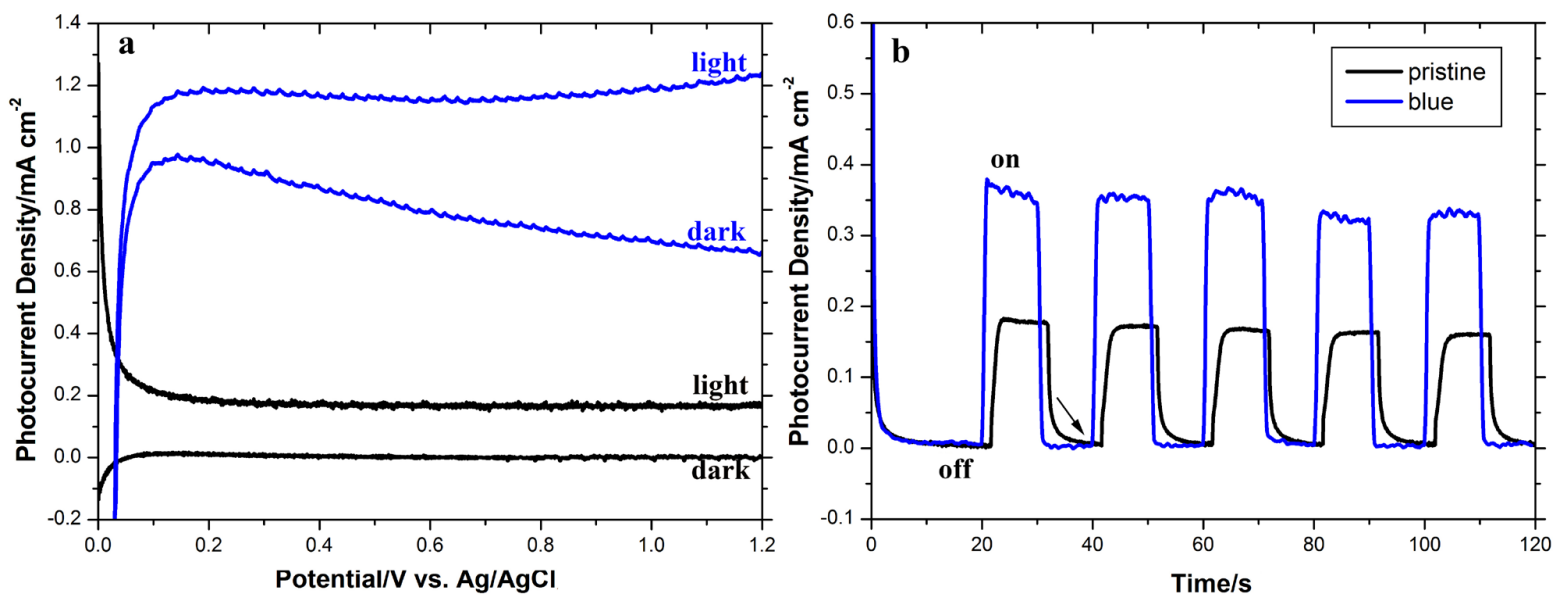
a) Linear sweep voltammograms collected under 100 mW cm^–2^ illumination at a scan rate of 100 mV s^-1^ and b) photoresponse vs. time under chopped light irradiation at fixed +0.5 V applied bias of the undoped (pristine) and blue TiO_2_NTAs.

To evaluate the self-doping process of a photoelectrochemical property, the photoactivity of pristine (undoped TiO_2_ NTAs annealed at 500 °C) and blue (annealed TiO_2_ NTAs reduced at –1.4 V for 10 min) TiO_2_ NTAs is compared in Figure 6. The LSV in the dark and under illumination is shown in Figure 6a. Unlike the pristine TiO_2_NTA with very low dark current, the dark current of blue TiO_2_NTA is greatly enhanced owing to increased carrier density and massive defects in the blue TiO_2_NTA. Also, clearly, the photocurrent of blue TiO_2_ NTAs under illumination is distinctly higher than that of the pristine, indicating more efficient charge separation and transport in the blue TiO_2_NTA owing to the high density of Ti^3+^ states. The photoresponses of the pristine and blue TiO_2_ NTAs measured by chronoamperometry are shown in Figure 6b. The photocurrent of blue TiO_2_NTA is about 2 times larger than that of pristine TiO_2_NTA because the blue TiO_2_ NTAs have a high amount of oxygen vacancy or Ti^3+^ donor sites, which leads to an increase in visible light absorption, charge separation, and transport of the sample. The results are comparable with similar studies in the literature [40,43,54,55]. Furthermore, as seen from Figure 6b, when the illumination changes to off, an apparent gradual decay of current (shown with arrow) could be seen in the pristine TiO_2_NTA, suggesting an onset of recombination in the semiconductor pristine TiO_2_ NTAs. In contrast, the blue TiO_2_NTA did not show this character and the photocurrent drastically decreased, confirming that the oxygen vacancies in the blue TiO_2_ NTAs act as charge carrier traps and hamper the electron-hole recombination [56].

**Figure 7:**
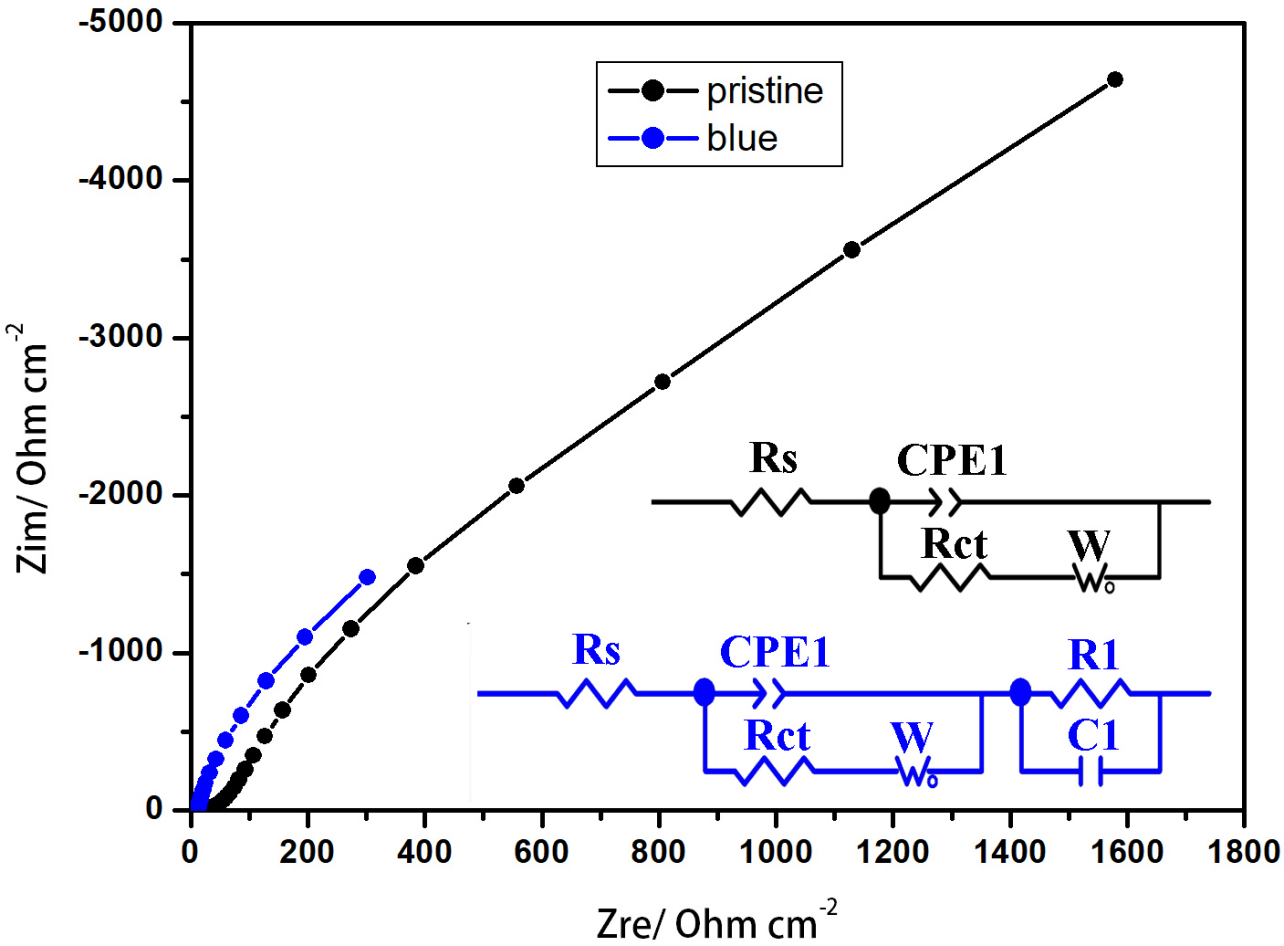
Nyquist representation of EIS data collected at OCP and equivalent circuit of the pristine and blue TiO_2_NTAs in 0.5 M Na_2_SO_4_.

To interpret the photoelectrochemical mechanism of the blue TiO_2_NTA, OCP and EIS measurements were carried out after inducing stable conditions via the supporting electrolyte of 0.5 M Na_2_SO_4_. Based on the results, open-circuit voltages of 42 and 65 mV were separately obtained for the pristine and blue TiO_2_NTA. Figure 7 depicts the Nyquist plot and the related equivalent circuit when the pristine and blue TiO_2_NTAwas placed in contact with the electrolyte. Rs is the resistance of the solution between the working and reference electrodes. Also, a serial combination of Faraday resistance and Warburg impedance is considered inside the nanotubes due to the diffusion-controlling nature of the process. In blue TiO_2_NTA, an RCpore connection was added in series to the equivalent circuit due to its capacitive behavior [26].

The straight line at 45° in Nyquist plots indicates diffusion processes in the system. The effect of blue TiO_2_NTA on the kinetics of the electrochemical processes can be interpreted from the data using ZView software. The ZView fitting results have been listed in Table 1. As seen from Table 1, the Rct resistance of the blue TiO_2_NTA was reduced to a negligible content of 0.000109 Ω cm^-2^. This phenomenon is interpreted by reducing the bandgap owing to the oxygen vacancy levels, so the diffusion becomes completely capacitive. DRS measurements on the blue TiO_2_ nanotubes revealed a significant reduction in bandgap energy. The results showed a narrowing band gap while doping manifesting a reduction of the bandgap from 3.16 eV for the pristine to 2.7 eV for the blue TiO_2_ NTAs. Therefore, the absorption wavelength shifted from 390 nm to around 460 nm, which is a shift to the inset of the visible region [26]. Flatband potentials from the Mott–Schottky curves can be estimated as 40 mV for the pristine TiO_2_NTA. A positive slope of the samples indicates the semiconducting behavior of the n-type electrodes. Pristine TiO_2_NTA displayed a strong capacity dependence on the voltage that is normal for the space charge layer-controlled capacity of an n-type semiconductor. In contrast, the blue TiO_2_NTA sample exhibited a weak dependence of capacitance on the applied voltage, meaning that the blue sample has metallic behavior. In metallic behavior, a capacitance is solely determined by the Helmholtz layer at the solid-liquid interface, hence a flatband potential cannot be expected [26]. Therefore, the oxygen vacancy levels significantly improved the electron transport of the blue TiO_2_ NTAs. Previous studies stated that by decreasing the resistance, an electron is easy to be transferred to the underlying TiO_2_, hence electron injection is enhanced and photo application of the electrode is improved [57].

**Table 1:** Calculated values of the equivalent circuit elements for the pristine and blue TiO_2_NTA.

Sample	Pristine	Blue
Rs (Ωcm-2)	10.51	8.19
CPE1-T (mFsp-1cm-2)	4.68 × 10–5	5.19 × 10–3
CPE1-P	0.56	0.79
Rct (Ωcm-2)	28.68	0.000109
W-R (Ωcm-2)	436.1	55.32
W-T	0.46	0.32
W-P	0.49	0.56
R1 (Ωcm-2)	-	401.3
C1 (mFsp-1cm-2)	-	0.060421

## 4. Conclusion

Self-doped (blue) TiO_2_ NTAs can be produced through a simple cathodic polarization method. Tuning the annealing temperature of TiO_2_ NTAs shows the high photoelectrochemical activity of TiO_2_ NTAs annealed at 500 °C. The photoelectrochemical activity of the blue TiO_2_ NTAs depends on the cathodic polarization parameters. Blue TiO_2_ NTAs synthesized at –1.4 V with a duration of 10 min exhibit twice more photocurrent response (0.39 mA cm^-2^) compared to the undoped TiO_2_ NTAs (0.19 mA cm^-2^). For blue TiO_2_ NTAs, oxygen vacancies decrease the charge recombination and enhance the charge transfer rate, consequently leading to high photoelectrochemical activity needed for water splitting applications.
